# Orange Judd upon Quackery

**Published:** 1874-04

**Authors:** 


A PARTY in New York, one David Richards, entered suit for damages against Orange Judd & Co., publishers of the *American Agriculturist*, for alleged *libel* in openly denouncing his so-called "Golden Remedies," and warning the public against them through the columns of their journal. We append a brief extract from the opinion of Judge Davis, of the Supreme Court, in dismissing the complainant with costs, it being of interest to medical readers:

"No one can read the circulars of the plaintiff, as proved by himself on his examination, without observing the importance of the investigation sought to be made. It was competent to disprove the assertions of the circulars and of the complaint by ascertaining the ingredients of the several compounds for the purpose of showing that they possess no such medical virtues as are claimed by plaintiff. For instance, he asserts in his circular that his 'Elixir of Love is composed of the most powerful ingredients of the vegetable kingdom—harmless, but speedy in restoring healthy action.' And again: 'It is the fountain of youth to old age, the rejuvenator of pristine vigor in the young; to the barren women of our land it is a special blessing.' Indeed, it is impossible to read the vulgar and, in many respects, shameful assertions and instructions that accompany the compounds of plaintiff without being struck with the vileness of the impostures. That he can bring an action of libel for injury alleged to be done to his trade in his medicines by denouncing them as arrant quackery, and at the same time protect himself against exposure by claiming them to be valuable secrets, is a proposition that cannot be maintained.

"In the laudable exposure of such humbugs as the pretended medicine of plaintiff and others, the defendants take upon themselves great risks, and subject themselves to the annoyance of suits; but I think they are not exposed to any danger that courts will interpose any shield for the protection of parties guilty of fraud and deception of the public."

In this connection we feel that it is due to Mr. Orange Judd to say that he has been for many years most laudably engaged in a crusade against all impostors and charlatans, and has done much to uphold the dignity and honor of the profession to which we belong, and to which we suspect he also belongs. We ought to say also that we have subscribed for and read the *American Agriculturist* for years, and esteem it to be unquestionably the *foremost agricultural journal* in this, or, as far as we know, in any other, country. Notwithstanding its low price ($1.50 per year,) it gives the largest quantity of the best agricultural reading of any similar periodical, and is only excelled by the *Hearth and Home*, from the same publishers, which, however, costs a dollar more. Our readers who desire agricultural or fireside reading cannot do better than to encourage Orange Judd in his fierce raid upon quackery and imposture, whilst at the same time making a good and cheap investment in the very best of reading matter and artistic engravings. A very handsome chromo is given free to each subscriber for each of the publications.

